# CD4-Binding Site Directed Cross-Neutralizing scFv Monoclonals from HIV-1 Subtype C Infected Indian Children

**DOI:** 10.3389/fimmu.2017.01568

**Published:** 2017-11-15

**Authors:** Sanjeev Kumar, Rajesh Kumar, Lubina Khan, Muzamil Ashraf Makhdoomi, Ramachandran Thiruvengadam, Madhav Mohata, Mudit Agarwal, Rakesh Lodha, Sushil Kumar Kabra, Subrata Sinha, Kalpana Luthra

**Affiliations:** ^1^Department of Biochemistry, All India Institute of Medical Sciences, New Delhi, India; ^2^Department of Pediatrics, All India Institute of Medical Sciences, New Delhi, India

**Keywords:** human immunodeficiency virus type-1, subtype C, CD4-binding site, RSC3 core protein, neutralizing antibodies, pediatric cross-neutralizers, scFv, phage display

## Abstract

Progression of human immunodeficiency virus type-1 (HIV-1) infection in children is faster than adults. HIV-1 subtype C is responsible for more than 50% of the infections globally and more than 90% infections in India. To date, there is no effective vaccine against HIV-1. Recent animal studies and human Phase I trials showed promising results of the protective effect of anti-HIV-1 broadly neutralizing antibodies (bnAbs). Interaction between CD4 binding site (CD4bs) on the HIV-1 envelope glycoprotein and CD4 receptor on the host immune cells is the primary event leading to HIV-1 infection. The CD4bs is a highly conserved region, comprised of a conformational epitope, and is a potential target of bnAbs such as VRC01 that is presently under human clinical trials. Recombinant scFvs can access masked epitopes due to their small size and have shown the potential to inhibit viral replication and neutralize a broad range of viruses. Pediatric viruses are resistant to many of the existing bnAbs isolated from adults. Therefore, in this study, pooled peripheral blood mononuclear cells from 9 chronically HIV-1 subtype C infected pediatric cross-neutralizers whose plasma antibodies exhibited potent and cross-neutralizing activity were used to construct a human anti-HIV-1 scFv phage library of 9 × 10^8^ individual clones. Plasma mapping using CD4bs-specific probes identified the presence of CD4bs directed antibodies in 4 of these children. By extensive biopanning of the library with CD4bs-specific antigen RSC3 core protein, we identified two cross-neutralizing scFv monoclonals 2B10 and 2E4 demonstrating a neutralizing breadth and GMT of 77%, 17.9 µg/ml and 32%, 51.2 µg/ml, respectively, against a panel of 49 tier 1, 2 and 3 viruses. Both scFvs competed with anti-CD4bs bnAb VRC01 confirming their CD4bs epitope specificity. The 2B10 scFv was effective in neutralizing the 7 subtype C and subtype A pediatric viruses tested. Somatic hypermutations in the VH gene of scFvs (10.1–11.1%) is comparable with that of the adult antibodies. These cross-neutralizing CD4bs-directed scFvs can serve as potential reagents for passive immunotherapy. A combination of cross-neutralizing scFvs of diverse specificities with antiretroviral drugs may be effective in suppressing viremia at an early stage of HIV-1 infection and prevent disease progression.

## Introduction

A vaccine for human immunodeficiency virus type-1 (HIV-1) is a global health priority. Attempts are underway in designing HIV-1 immunogens that can elicit correlates of protection (1, 2). Using the reverse vaccinology approach, broadly neutralizing antibodies (bnAbs) are being used to map the neutralizing determinants that can be incorporated into an immunogen based vaccine that will be effective in eliciting similar antibodies in natural infection (2, 3). Majority of the bnAbs isolated so far are from non-subtype C infected individuals and except a few, not much effective against subtype C viruses (4–8).

The CD4 receptor on host immune cells is the primary receptor for HIV-1 entry and infection. The CD4-binding site (CD4bs) is a highly conserved, conformational, and discontinuous region in the envelope of HIV-1, HIV-2, and also in SIV (9–12). An anti-CD4bs bnAb VRC01 was isolated from a subtype B infected slow-progressor by constructing a resurfaced stabilized core protein, the best antigen designed so far to identify bnAbs against CD4bs region (13). Further, a number of bnAbs of the VRC01 class have been generated, suggesting that the CD4bs region is a prime target for bnAbs with high potency and breadth (14–16). In recent Phase I clinical trials, passive infusion of anti-CD4bs bnAbs 3BNC117 and VRC01 demonstrated reduced viremia in HIV-1-infected donors (17–22), suggesting that bnAbs directed against the CD4bs can serve as potential candidates for immunotherapy and to guide immunogen design.

Subtype C HIV-1 is the most predominant subtype circulating in India (23–26). Disease progression is faster in HIV-1 infected children than adults due to immaturity of the immune system in terms of both innate and acquired immune responses and limited exposure to diverse pathogens (27–29). Early initiation of antiretroviral therapy (ART) can prevent viral replication and delay disease progression, however, it cannot prevent HIV-1 infection (30). Most of the bnAbs isolated till date, are from adults, except a recently isolated bnAb BF520.1 from an HIV-1 subtype A infected infant demonstrating for the first time that cross-neutralizing antibodies (cnAbs) are elicited in infants too (31). A major concern is that the bnAbs isolated from non-subtype C infected adult donors when tested against pseudoviruses generated from the infected children were found less effective against HIV-1 subtype C viruses (32). Recently, we observed that majority of the viruses circulating in chronic HIV-1 infected children were resistant to neutralization by the second-generation anti-HIV-1 bnAbs isolated from adult donors (33). Hence, there is a need for the generation of anti-HIV-1 cnAbs from subtype C infected children that are effective against subtype C viruses, responsible for more than 50% of the global HIV-1 infection.

Broadly neutralizing antibodies are produced in 15–25% of the infected individuals after a minimum of 2–3 years of HIV infection (34). Previously, we have for the first time showed the presence of cnAbs targeting multiple HIV-1 epitopes in the plasma of HIV-1-infected children (35, 36). Later, the development of bnAbs in infants was reported by Goo et al. (37) followed by successful isolation of a bnAb BF520.1 directed against N332 V3-glycan epitope from an infant (31). In a recent study, we observed the evolution of cnAbs with multiple epitope specificities in the plasma of chronically infected pediatric donors (38) who were anti-retroviral naïve for ≥5 years of infection and potential long-term non-progressors, identifying them as suitable candidates for the isolation of bnAbs. The CD4bs on the viral envelope glycoprotein gp120 is a highly conformational epitope. Single chain antibody fragments (scFvs) retain their antigen binding sites and yet are small and flexible to overcome such constraints posed by conformational embedded epitopes. Phage display has proved to be a powerful method for the generation of recombinant antibody fragments (39, 40), its main advantage being the large diversity of antibodies with varied epitope specificities that can be generated by biopanning with different antigens (41–44). Therefore, this study was aimed to construct a peripheral blood mononuclear cell (PBMC) based scFv phage library, from nine subtype C infected antiretroviral naïve pediatric cross-neutralizers, whose plasma antibodies exhibited potent and cross-neutralizing activity, to identify scFvs against the CD4bs region of subtype C HIV-1. The phage library comprised of 9 × 10^8^ clones and further biopanning this library using CD4bs specific antigen RSC3 core protein, led to the identification of two cross-neutralizing anti-CD4bs scFv monoclonals. The pediatric anti-CD4bs scFv monoclonal 2B10 exhibited cross neutralization activity against subtype C viruses of Indian and African regions and with other non-subtype C viruses.

## Materials and Methods

### Study Subjects

Nine ART naïve HIV-1 subtype C chronically infected pediatric cross-neutralizers (AIIMS_329, AIIMS_330, AIIMS_341, AIIMS_346, AIIMS_355, AIIMS_357, AIIMS_505, AIIMS_509 and AIIMS_510) were recruited for this study from the Pediatric OPD, Department of Pediatrics, AIIMS, New Delhi. 5 ml of blood was drawn by venipuncture under aseptic conditions from all the study subjects. Blood was used for PBMC isolation after separating the plasma by centrifugation at 600 × *g*. The plasma was heat inactivated at 56°C for 1 h and stored at −80°C for further experiments.

### Antigens, Antibodies, and Vectors

The plasmids for envelope glycoprotein expression and the purified proteins used in this study were obtained from various sources; RSC3 core wild-type (WT) and its mutant Δ371I/P363N expression plasmids and proteins (kindly provided by Dr. John R. Mascola, NIAID, VRC, USA), HXBc2 WT and its D368R mutant gp120 plasmids (kindly provided by Dr. Joseph Sodroski, Dana Farber Cancer Institute, USA). The anti-HIV-1 bnAbs 2G12, VRC01 were obtained from the NIH AIDS Reagent Program (NIH ARP). Antibody 1418, specific for the capsid of human parvovirus B19, was kindly provided by Dr. Susan Zolla Pazner, NYU, USA. The plasmids for CAP256 and its N332A mutant were kindly provided by Dr. Lynn Morris, National Institute for Communicable Diseases, South Africa. The pAK100 and pAK400 plasmids were kindly gifted by Dr. Andreas Pluckthun, Department of Biochemistry, University of Zurich, Switzerland.

### Plasma Antibody Binding Analysis by ELISA

All antigens were coated on ELISA plates at a concentration of 2 µg/ml and incubated overnight at 4°C. Plates were blocked with 300 μl/well of 15% FBS in RPMI for 1.5 h, followed by addition of 100 μl/well of heat inactivated plasma at different dilutions (1:30, 1:100, 1:300, 1:1,000, 1:3,000, and 1:10,000) or anti-HIV-1 monoclonal antibodies (VRC01, b12, 447-52D and 2F5) and anti-Parvovirus mAb 1418, each at 10 µg/ml concentration and incubated for 1 h at 37°C. Each of the above steps was followed by washing with 1× PBS (Phosphate Buffered Saline) with 0.1% Tween 20. Next, 100 µl of alkaline phosphatase (AP)-conjugated anti-human IgG Fc antibody [1:2,000 diluted in 1× PBS with 0.05% Tween 20 (Southern Biotech)] was added to the plates and the immune complexes were reacted with AP substrate in 10% DAE buffer (1 mg/ml). Reaction was stopped by adding 6 N NaOH and plates were read at 410 nm. The reported Max50 value is the 50% maximal binding of plasma antibodies that showed saturation. Max50 values were calculated as described previously (45).

### Pediatric Anti-HIV-1 C scFv Phage Library Construction

A pediatric anti-HIV-1 subtype C scFv recombinant phage library was constructed as described previously (43, 44, 46, 47) with a few modifications. Briefly, one million PBMCs from each of the nine select pediatric cross-neutralizers were pooled and total RNA was isolated by Trizol reagent (Sigma) and then reverse transcribed to cDNA, using the Reverse aid M-MuLV reverse transcriptase (Thermo). For construction of scFv, the heavy chain and light chain variable region genes were amplified using specific primers (IDT) (Table S2 in Supplementary Material) (46) and hot start Taq DNA polymerase (Fermentas, USA). An equimolar mixture of pooled heavy and light chain DNA was used in the second round of assembly PCR using Pfu DNA polymerase. Full length scFvs were amplified by pull-through PCR reaction using Hot start Taq DNA polymerase and forward primer PTFw 5′ CCT TTC TAT GCG GCC CAG CCG GCC ATG GCC 3′ and reverse primers PAK kappa *Sfi* 5′ TCA GCA TGG CCC CCG AGG CCG CAC GTT TRA T 3′, and PAK lambda *Sfi* 5′ TCA GCA TGG CCC CCG AGG CCG CAC CTA RRA C 3′ (R = G and A). The scFvs were resolved on agarose gel and purified using gel extraction kit (Qiagen). The library was constructed by ligating the scFv into pAK100 phagemid vector by using T4 DNA ligase (New England Biolabs) followed by transformation into TG1 electrocompetent cells by electroporation (current 25°F, resistance 200 Ohms, voltage 2,500 V) (BioRad). The transformed cells were plated on to 2XYT medium agar plates containing chloramphenicol (30 µg/ml) and incubated overnight at 37°C. A glycerol stock of the recombinant scFv library was made and aliquots stored at −80°C.

### Colony PCR and scFv Sequence Analysis

Twenty scFv clones were randomly picked from the library to check the presence of scFv inserts and diversity of the scFv phage library. Colony PCR was done by using forward primer; PTfw 5′ CCT TTC TAT GCG GCC CAG CCG GCC ATG GCC 3′ and reverse primer; (pAK *Sfi*1) 5′ TCA GCA TGG CCC CCG AGG CCG CAC GTT TRAT 3′, PAK lambda *Sfi* 5′ TCA GCA TGG CCC CCG AGG CCG CAC CTA RRA C 3′ (R = G and A). Plasmid DNA of colony PCR positive scFv clones was isolated, sequenced commercially by Macrogen (South Korea), and the sequences were analyzed by online IMGT/V-Quest software provided by the international ImMunoGeneTics database (IMGT) (http://www.imgt.org/IMGT_vquest/share/textes/).

### Biopanning of the scFv Phage Library

The rescue of phage library was done with M13KO7 helper phage (Stratagene) as mentioned previously (44, 48). The phages were then subjected to five rounds of enrichment by bio-panning as described earlier (43). Briefly, RSC3 core protein was coupled to magnetic beads (MyOne Tosyl activated Dynalbeads, Invitrogen) according to the manufacturer’s instructions, and the antigenic integrity of protein after bead coupling was verified by flow cytometry, using bnAb VRC01. Phages were transferred to an eppendorf tube containing 60 µl RSC3 core protein coated magnetic beads and incubated for 1 h at RT with gentle shaking. The unbound phage was removed by washing 10–15 times with 1× PBS with 0.1% Tween 20. The bound phage was eluted with 0.2 M glycine pH 2.2 for 10 min at RT. The eluted phage were neutralized with 1 M Tris-HCl pH 9.2 and immediately added to TG1/HB2151 cells (OD = 0.5) for infection at 37°C without shaking for 30 min and with shaking at 37°C for 30 min. Cells were spun down and plated on 2XTY agar containing chloramphenicol (30 µg/ml). Finally, the individual colonies were picked and grown in 96 well sterile culture plates (Corning) and a glycerol stock of each colony was stored at −70°C. This procedure was again repeated four times to complete five rounds of biopanning.

### Soluble Phage ELISA

Soluble phage ELISA was performed as described previously (43, 49, 50), with few modifications. The ELISA plates were coated with 100 µl of RSC3 core and its mutant Δ371I/P363N at 2 µg/ml in 0.1 M NaHCO_3_ (pH 8.6) and incubated overnight at 4°C. The anti-CD4bs bnAbs VRC01 and b12 was used as positive controls; anti-CD4bs non-neutralizing antibody (non-nAb) b6, anti-V3 mAb 447-52D, anti-Parvovirus mAb 1418, and scFv against hepatitis virus HepB scFv were used as negative controls at 1 µg/ml concentration in these assays. The phage clones were taken as positive by ELISA, if the absorbance of RSC3 core coated wells was at least two times higher than RSC3 core mutant delta 37I/P363N and at least three times higher than binding of phage clones to unrelated antigens (unrelated peptide pool, BSA).

### Sequencing Analysis of scFvs

Sequencing analysis of positive scFv clones of the soluble phage ELISA was performed as described previously (43, 44). Briefly, the plasmid of positive scFv clones from the soluble phage ELISA was isolated by using plasmid purification Mini kit (Qiagen, Germany) and sequenced commercially by Macrogen (South Korea). The sequences were analyzed using IMGT V Quest software.

### Expression and Purification of scFv Monoclonals

The binding clones from the soluble phage ELISA were selected for soluble scFv expression as described earlier (43). The pAK100 vector carrying scFv DNA and pAK400 vector were digested with *SfiI* restriction enzyme (New England Biolabs). The digested scFv DNA fragments and pAK400 vector were gel purified using gel extraction kit (Qiagen). The scFv DNA was ligated into pAK400 vector at a 3:1 M ratio using T4 DNA ligase (New England Biolabs) in a reaction volume of 20 µl followed by transformation into HB2151 cells using calcium chloride method. The HB2151 cells carrying scFv cloned pAK400 plasmid were grown and induced by 1 mM IPTG for 4 at 24°C (48). Next, scFv was purified from the periplasmic extract by Ni-NTA (Qiagen) affinity chromatography as described earlier (44). The protein was eluted with 300 mM imidazole followed by extensive dialysis against ice cold 1× PBS (pH 7.4), and protein samples were concentrated using ultrafiltration columns (Ambion) over a 10 kDa cut off and filtered by 0.2 µm sterile syringe filter. The purity of the purified proteins was assessed by SDS-PAGE and Western blot. Briefly, resolving the scFv on 12% SDS-PAGE followed by Western blotting, the scFv was detected using anti-His-tag antibody raised in mouse (Sigma) at a 1:1,000 dilution in 1× PBS with 0.05% Tween 20. Secondary anti-mouse HRP (1:3,000 dilutions) antibody was added and incubated at RT for 2 h, and color was developed using 3,3′-diaminobenzidine tetrahydrochloride (DAB) as substrate. Proteins with >95% purity were stored at −80°C immediately after snap freezing in liquid nitrogen.

### Purified scFv ELISA and Affinity Determination

ELISA of purified scFvs was done by using HIV-1 monomeric and trimeric envelope glycoproteins (HIV-1 96ZM651 gp120 monomer subtype C, BG505 SOSIP.664 D7324 trimeric antigen and HxBc2 gp120 and HxBc2 D368R mutant gp120 monomers) as described previously (2, 43). Briefly, all antigens were coated on ELISA plates at 2 µg/ml concentration, blocked with 15% FBS, 2% BSA in a RPMI media; threefold serial dilutions of scFvs and mAb controls (VRC01, b12, 447-52D, 2G12, 2F5 and 1418) were added followed by detection with 1:1,000 dilution of primary antibody (Anti-His tag for scFvs and HRP conjugated anti-human Fc antibody for mAb controls) in 2% milk phosphate-buffer saline (MPBS). For scFvs, 1:2,000 diluted anti-mouse HRP-conjugated secondary antibody in 2% MPBS was added. Each of the above steps was followed by washing the plate with 1× PBS with 0.1% Tween 20. Immediately after washing, 100 µl of TMB substrate (BioLegend) was added and incubated at RT till the color developed. Reaction was stopped by adding 8 N H_2_SO_4_ and the absorbance was read at 450 nm.

Affinity analysis of the scFvs was done by ELISA as described previously (43). Briefly, different dilutions of BG505:SOSIP.664 gp140 (10–800 nM) were incubated with fixed concentration of the scFvs (3.125 nM) for 16 h at 4°C. Next day, 100 µl of the equilibrated solution was incubated with BG505:SOSIP.664 gp140 coated ELISA plates (500 ng/well) for 20 min at RT to capture the free scFvs. Bound scFv was detected with primary anti-His tag antibody (raised in mouse, Sigma) at 1:1,000 dilution and with secondary anti-mouse HRP conjugated antibody (Sigma) at 1:2,000 dilution as described before. Saturation binding curve was plotted using non-linear regression method and KD values were calculated. Competition ELISA was performed as described in Ref. (13, 43) by using biotinylated VRC01 and BG505:SOSIP.664 gp140 antigen. All the ELISAs were repeated twice and data was analyzed by using GraphPad Prism software 5.0.

### Viral Neutralizing Activity of scFvs

The neutralization efficiency of the purified scFv monoclonals was tested with HIV-1 pseudoviruses and primary isolates as described previously (43, 44, 51). Briefly, HIV-1 envelope expression plasmids from a standard panel for subtypes A, B, C, AD, CD, and CRF2 AG were obtained from the NIH AIDS Reagent Program (NIH ARP). Pseudoviruses were generated by co-transfecting HEK293T cells with a plasmid expressing envelope and envelope deficient HIV-1 backbone vector pSG3Δenv as described earlier (51), and primary isolates were derived from PBMCs of HIV-1 infected Indian donors as reported earlier (33, 45, 52). Next, starting with 100 µg/ml of scFv concentration, three-fold serial dilutions were made in 10% DMEM [Dulbecco Modified Eagles’s Medium, HyClone, GE Healthcare supplemented with 10% FBS (Fetal Bovine Serum) and 1% PenStrep (Penicillin and Streptomycin) antibiotics] and incubated with 200 TCID of the pseudoviruses/primary isolates for 1 h at 37°C. Then, 10^4^ cells/well TZM-bl cells were added to the scFv and virus complex along with 25 µg/ml DEAE Dextran (Sigma) (Indinavir (1 mM) was additionally added with primary isolates). After 48 h, 150 µl volume of culture medium was removed and 50 µl of Bright-Glo Luciferase assay reagent (Promega) was added to TZM-bl cells followed by 2-min incubation at room temperature to allow complete cell lysis. Then, luciferase activity was read in 96-well black plate (Costar) using Synergy 2 Multi-Mode Reader (BioTek). The IC50 values for the scFvs were calculated by a dose-response curve fit with non-linear function, with the help of Graph Pad prism software 5.0. Each experiment was repeated twice and performed in duplicates and IC50 titers were calculated.

### Statistical Analyses

Statistical analyses were performed using Graph Pad Prism 5. Non-linear regression curve straight line was plotted using the method of least squares to determine the Max50 and IC50 values. Mean Max50 binding titers were compared using unpaired *t* test. *P* values <0.05 were considered significant.

## Results

### Plasma Samples of HIV-1 Infected Pediatric Donors Showed the Presence of Anti-CD4bs Antibodies

**Figure 1:**
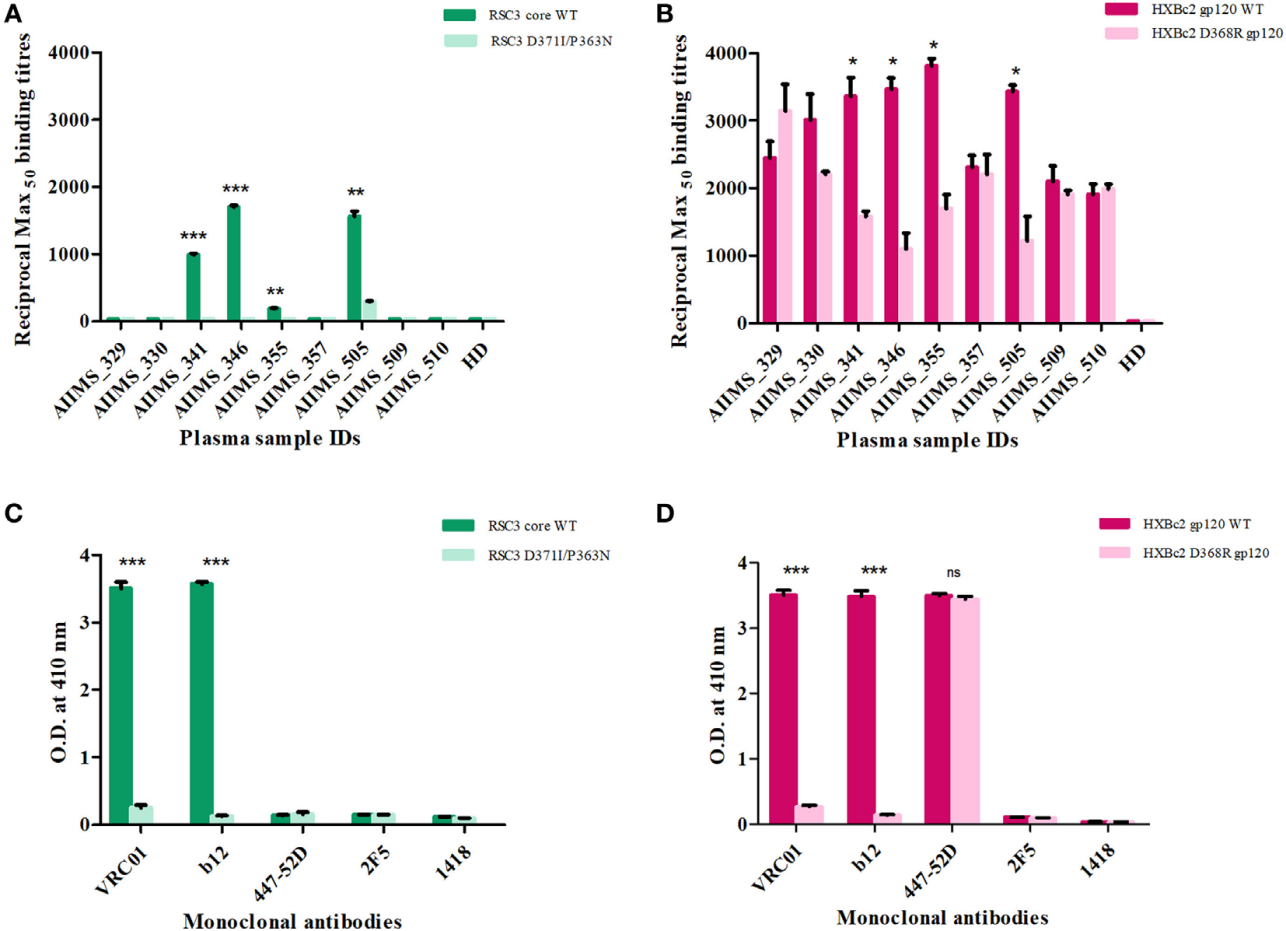
Screening of cross-neutralizing plasma (CNP) samples for the presence of anti-CD4 binding site (CD4bs) antibodies. **(A)** Nine CNP samples were tested for the presence of anti-CD4bs antibodies using CD4bs specific probes RSC3 core wild-type (WT) and its mutant Δ371I/P363N at 2 µg/ml. **(B)** CNP samples were further tested for the presence of anti-CD4bs antibodies using HXBc2 WT and its mutant HXBc2 D368R gp120. Reciprocal Max50 binding titers were calculated using the least square regression method. The ELISA was repeated twice in triplicates. HD was the plasma of one healthy seronegative donor used as negative control. Mean reciprocal Max_50_ binding titers were compared using unpaired *t*-test for each plasma sample for RSC3 core WT and its mutant or HXBc2 WT and its mutant D368R. *P* values <0.05 were considered significant and shown by asterisk (*) symbol, (**) if *p* < 0.01 and (***) if *p* < 0.001. **(C)** The binding of RSC3 core WT and its mutant Δ371I/P363N was checked with anti-CD4bs mAb VRC01 and b12 (positive controls) and anti-V3 mAb 447-52D, anti-MPER mAb 2F5 and anti-Parvovirus mAb 1418 were used as negative controls. **(D)** The binding of HXB2 gp120 WT and its D368R mutant was tested with mAbs.

Nine anti-retroviral naïve HIV-1 subtype C infected pediatric cross-neutralizers were recruited for the generation of the human anti-HIV-1 scFv recombinant library, based on the viral cross neutralizing potential exhibited by their plasma antibodies (35, 36, 38). The demographic and clinical profile of the study subjects is provided in Table 1. The infected donor plasma samples were analyzed for the presence of anti-CD4bs antibodies using recombinant gp120 proteins (13, 53) known to bind CD4bs-directed bnAbs and their corresponding mutants; the RSC3 core protein and its mutant Δ371I/P363N, HXBc2 WT gp120 and its mutant D368R. The plasma antibodies of 4 pediatric donors (AIIMS_341, AIIMS_346, AIIMS_357, and AIIMS_361) demonstrated high binding titers with RSC3 core WT protein as compared to its mutant Δ371I/P363N, suggesting the presence of VRC01 like anti-CD4bs antibodies in their plasma (Figure 1A); this was further confirmed with HXBc2 WT gp120 and its mutant D368R (Figure 1B). The results for the binding of positive and negative control antibodies with RSC3 core WT and its mutant Δ371I/P363N are shown in (Figure 1C) and with HXBc2 WT and its mutant D368R in (Figure 1D).

**Table 1:** Clinical and demographic profile of 9 chronically HIV-1 infected children at the time of enrollment in study.

Pediatric donor ID	Age (in years)/gender	HIV-1 subtype	Viral load (RNA copies/ml)	CD4 count (cells/μl)
AIIMS_329	6/M	C	39,000	1,280
AIIMS_330	6/M	C	27,500	1,174
AIIMS_341	5/M	C	NA	NA
AIIMS_346	8/M	C	73,900	441
AIIMS_355	11/M	C	48,300	850
AIIMS_357	12/M	C	19,200	510
AIIMS_505	5/F	C	34,300	1,128
AIIMS_509	8/M	C	3,410	918
AIIMS_510	8/F	C	11,500	676

### Anti-CD4bs Specific scFv Clones Identified in the Pediatric Anti-HIV-1 scFv Recombinant Phage Library

**Figure 2:**
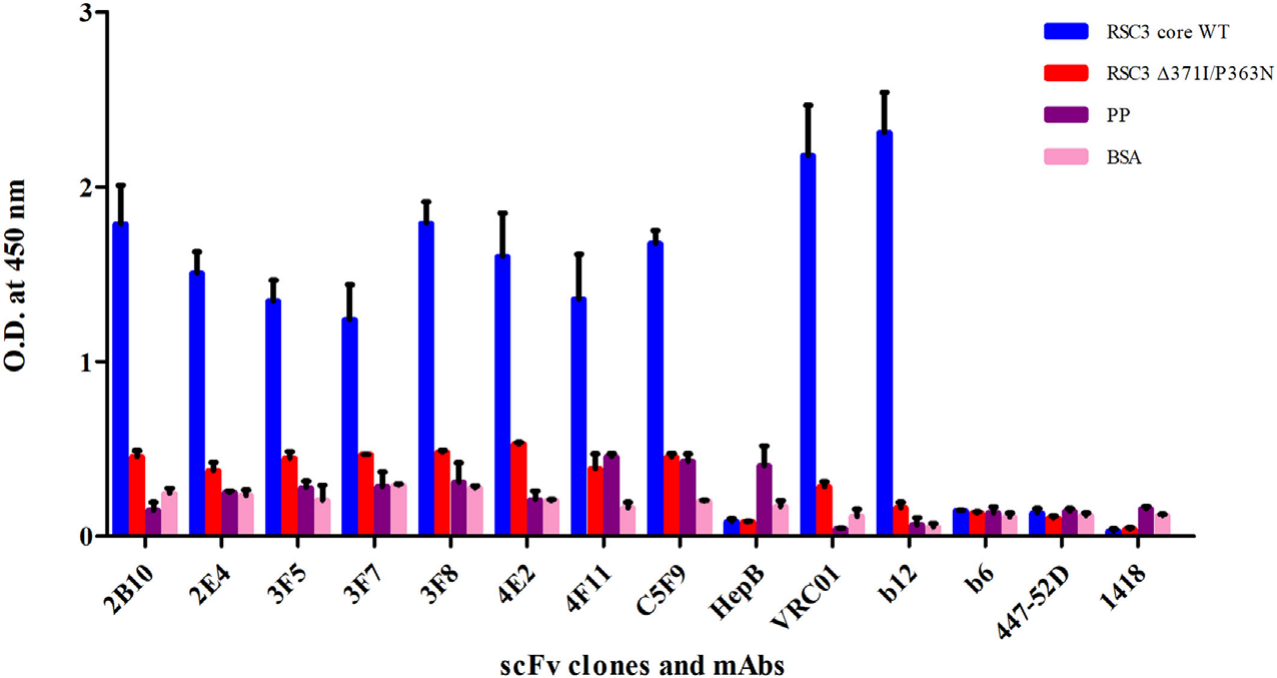
Soluble phage ELISA for the identification of anti-CD4-binding site (CD4bs) specific scFv clones. RSC3 core WT along with its mutant Δ371I/P363N and negative control antigens PP-unrelated peptide pool and BSA was used at 2 µg/ml. Here, anti-CD4bs bnAbs VRC01 and b12 were used as positive controls; anti-CD4bs non-neutralizing antibody (non-nAb) b6, anti-V3 mAb 447-52D and anti-parvovirus mAb 1418 were used as negative controls along with anti-Hepatitis HepB scFv clone. The scFv Clones showing binding with RSC3 core WT antigen at least two times more than its mutant and three times than the negative control were considered as positive. The ELISA was repeated twice and mean binding titers were compared with negative controls.

The PBMCs from 9 pediatric cross-neutralizers were pooled and an anti-HIV-1 pediatric scFv phage library was constructed, as described in the methodology section. The variable genes for heavy chain (VH) and light chains kappa and lambda (VLκ and VLλ) were successfully amplified showing desired 400 bp bands, except VLκ3 and VLλ2 (Figures S1A–C in Supplementary Material) followed by successful construction of scFv gene (800 bp) by pull-through PCR (Figure S1D in Supplementary Material). Next, colony PCR of 20 random clones from the unselected phage library (before biopanning) showed two clones with no scFv inserts (Figure S2 in Supplementary Material) thus, confirming 90% ligation efficiency. DNA sequencing of 18 colony PCR positive clones confirmed that sequence of all scFv inserts were distinct (Table S1 in Supplementary Material). On the basis of these results, it is inferred that, diversity of the constructed phage library was 90% with 9 × 10^8^ diverse clones. Biopanning of the library was performed using RSC3 protein coated on magnetic beads. The integrity of coated RSC3 protein on the beads was checked by flow cytometry by using anti-CD4bs bnAb VRC01 and more than 99% beads were found coated with RSC3 core protein (Figure S3 in Supplementary Material). After five rounds of biopanning, 150 scFv clones were randomly selected, their binding specificity was checked and eight scFv clones were identified which showed highest binding with the RSC3 protein and a decrease in binding of at least twofold with RSC3 Δ 371I/P363N protein, demonstrating CD4bs specificity (Figure 2).

### Sequence Analysis of Identified scFv Clones Showed Distinct Characteristics

The plasmid of eight soluble phage ELISA positive scFv clones was isolated and sequenced, and we found 7/8 clones were similar in sequence because of the enrichment of antigen-specific clones after five rounds of biopanning. Therefore, two clones (2B10 and 2E4) out of eight identified scFv clones were distinct and were taken for further analysis. Analysis of the gene usage of the scFvs by IMGT/V-Quest software program revealed that both 2B10 and 2E4 scFvs are variants of the same immunoglobulin heavy chain gene family IGHV1-02*02, but having different D and J region alleles, i.e., IGHD6-19*01 and IGHJ4*02 for 2B10 and IGHD6-13*01 and IGHJ3*01 for 2E4. The CDRH3 and CDRL3 region of 2B10 is shorter (13 amino acids in CDRH3 and 12 amino acids in CDRL3) than CDRH3 and CDRL3 regions of 2E4 (18 amino acids in CDRH3 and 13 amino acids in CDRL3). Also, higher number of somatic hypermutations (SHMs) was present in the immunoglobulin heavy chain and light chain genes of 2B10 than 2E4 (Table 2).

**Table 2:** The heavy and light chain gene sequence analysis of identified anti-CD4bs scFv clones.

Heavy chain						

scFv ID	V-gene	D-gene	J-gene	CDRH3	CDR3 length (amino acids)	VH nucleotide mutation frequency
2B10	IGHV1-2*02	IGHD6-19*01	IGHJ4*02	CARGDSSGWYGFDF	13	30/295 (10.1%)
2E4	IGHV1-2*02	IGHD6-13*01	IGHJ3*01	CARERVPYGSSWYNDAFDVW	18	33/296 (11.1%)

**Light chain**						

**scFv ID**	**V-gene**	**J-gene**		**CDRL3**	**CDR3 length (amino acids)**	**VH nucleotide mutation frequency**

2B10	IGLV1-51*02	IGLJ2*01		CGTWDSSLSAVVF	13	09/293 (3.03%)
2E4	IGLV3-19*01	IGLJ2*01		CNSRDSSGNHLEF	13	0/290 (0%)

*The genes of two scFv monoclonals 2B10 and 2E4 were sequenced commercially and their sequence was analyzed by using IMGT/V-Quest software*.

### The Pediatric scFv Monoclonals 2B10 and 2E4 Showed CD4bs Epitope Specificity in Binding Assay Confirmed by Competitive ELISA

**Figure 3:**
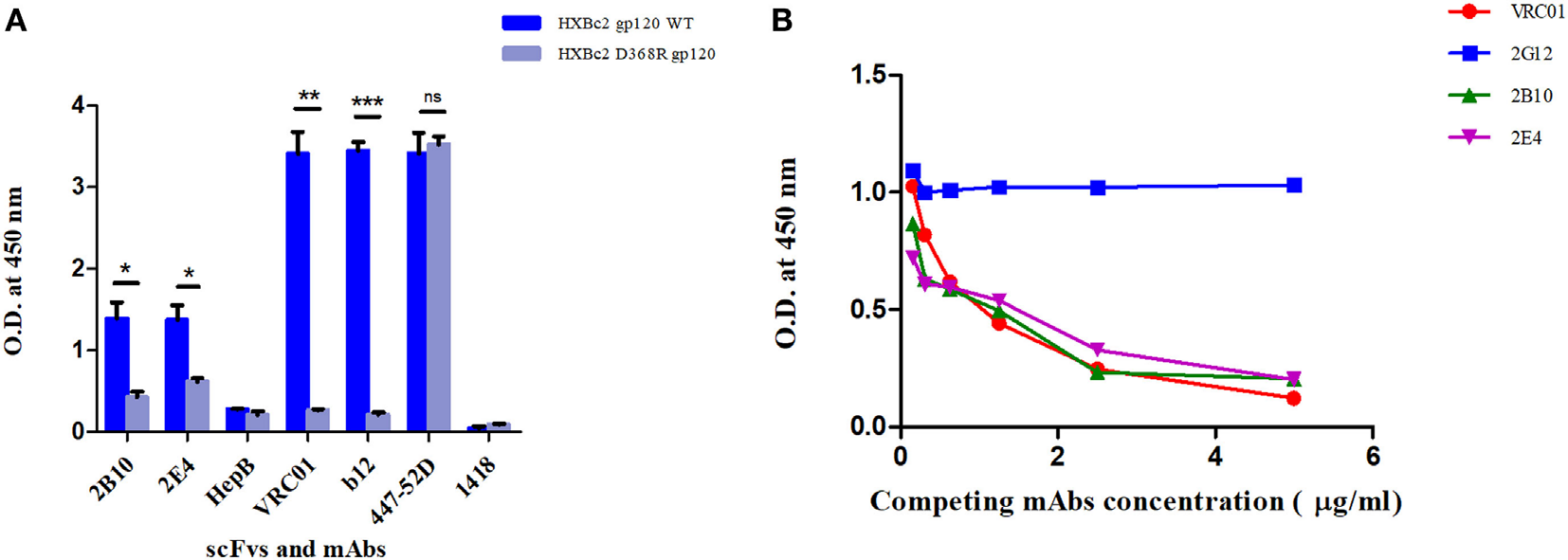
ELISAs to determine epitope specificities of scFv monoclonals. **(A)** Purified scFv monoclonals were checked for binding reactivity with HXB2 gp120 WT and its D368R mutant by ELISA. Here, anti-HIV-1 bnAbs VRC01 and b12 were used as positive controls and anti-V3 mAb 447-52D, anti-Parvovirus mAb 1418 with anti-hepatitis scFv HepB were used as negative controls. Mean binding titers were compared using unpaired *t*-test. *P* values < 0.05 were considered significant and shown by asterisk (*) symbol, (**) if *p* < 0.01 and (***) if *p* < 0.001. Here, ns is designated for “not significant.” **(B)** Competition ELISA of biotinylated VRC01 (at fixed concentration of 50 ng/ml) to BG505:SOSIP.664 D-7324 with decreasing concentrations of scFvs (2B10 and 2E4) and mAbs (2G12, VRC01). 2G12 bnAb was used as negative control, and the bnAb VRC01 was used as positive control for the assay. Dilution used for antibodies was 5 µg/ml to 0.1526 µg/ml.

The SDS-PAGE and Western blot analysis of the purified scFv clones 2B10 and 2E4 confirmed that both scFv proteins were more than 95% pure and showed the presence 32 kDa scFv bands (Figure S4 in Supplementary Material). The purified 2B10 and 2E4 scFvs were tested further in ELISA binding assays using HXBc2 gp120 and its mutant D368R. Both the scFvs showed binding to HXBc2 gp120 protein, with no significant binding to its corresponding HXBc2 D368R mutant, as is expected of CD4bs directed Abs (Figure 3A). This was further confirmed by competition ELISA with biotinylated VRC01 (Figure 3B). Moreover, both scFv monoclonals did not show any binding with V3 and MPER peptides (data not shown). In addition, the neutralization activity of both the scFv monoclonals was assessed for their V1V2 and V3-glycan reactivity by using CAP256 WT and its V3-glycan N332A mutant pseudoviruses (Table 3) and 16055 WT and its V1/V2 mutants N156K, N160K, K169A, and K171A pseudoviruses (Table 4). We observed, neither of the two scFvs exhibit V1/V2 or V3-glycan reactivity.

**Table 3:** Assessment of N332 V3-glycan dependent neutralization activity of 2B10 and 2E4 scFvs.

	IC50 titers (μg/ml)		Fold change
scFv ID	CAP256 WT	CAP256 N332A	N332A
2B10	39.7	37.4	0.94
2E4	90.2	88.9	0.98

*The neutralization reactivity with V3-glycan N332 of the identified scFv monoclonals 2B10 and 2E4 was assessed by using human immunodeficiency virus type-1 subtype C pseudoviruses CAP256 wild-type and its mutant N332A using a starting concentration of 100 µg/ml of scFvs. Neither of the two scFv monoclonals shows N332A dependent neutralization activity*.

**Table 4:** Assessment of V1V2 dependent neutralization activity of 2B10 and 2E4 scFvs.

	IC50 Titers (μg/ml)					Fold change			
scFv ID	16055 WT	N156K	N160K	K169A	K171A	N156K	N160K	K169A	K171A
2B10	35.5	34.3	33.78	32.4	34.9	0.96	0.95	0.91	0.98
2E4	81.2	80.3	80.5	79.6	74.6	0.98	0.99	0.98	0.91

*The neutralization reactivity with V1V2 region mutants of the 2B10 and 2E4 scFvs were performed using HIV-1 subtype C pseudovirus 16055 and its V1V2 mutants N156K, N160K, K169E, and K171A using a starting concentration of 100 µg/ml of scFvs. Neither of the two scFv monoclonals shows V1V2-dependent neutralization activity*.

### Binding Reactivity and Affinity Measurement of the Pediatric scFv Monoclonals 2B10 and 2E4 to Trimeric and Monomeric gp120 Envelope Glycoprotein Confirms Their Binding to the Native Viral Envelope

**Figure 4:**
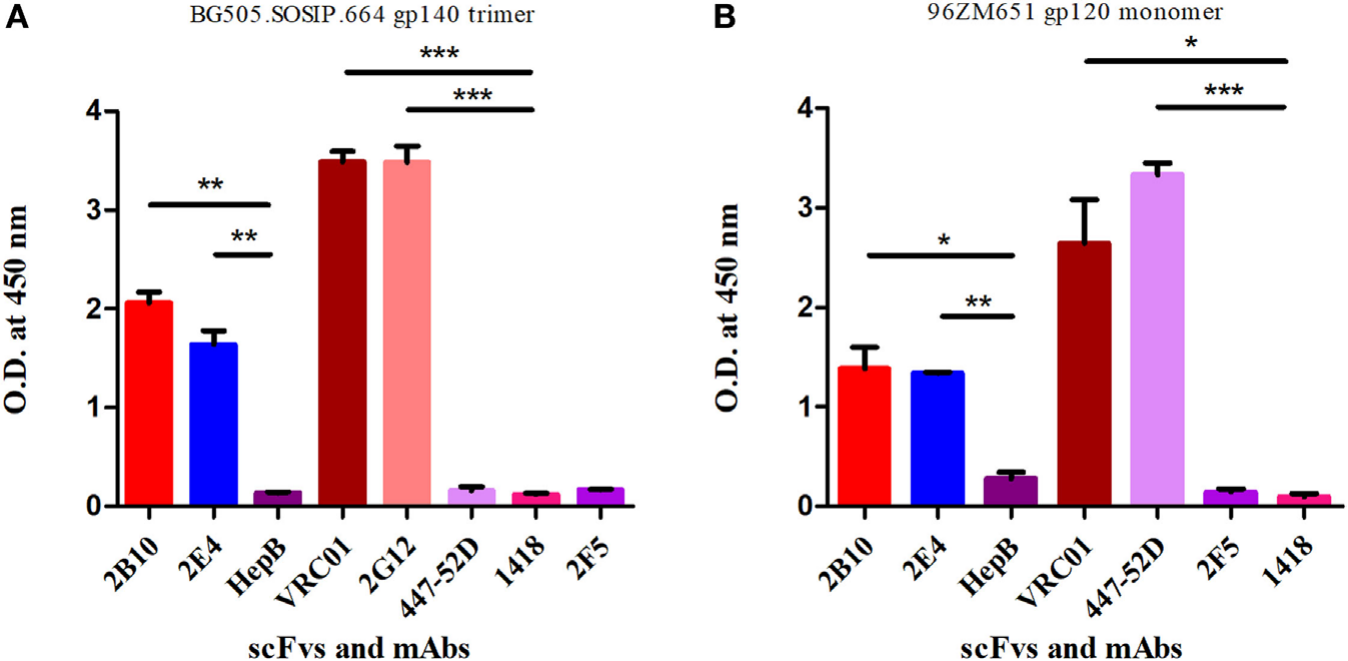
ELISA binding reactivity of 2B10 and 2E4 scFvs with HIV-1 monomeric and native-like trimeric envelope glycoproteins. ELISA was done to determine binding reactivity of the scFvs with **(A)** native-like HIV-1 BG505-SOSIP.664 D-7324 gp140 trimeric protein at 2 µg/ml. The anti-HIV-1 mAbs VRC01 and 2G12 were used as positive controls and anti-HIV-1 gp41 (MPER) 2F5, anti-V3 mAb 447-52D, anti-parvovirus mAb 1418, and anti-hepatitis scFv HepB were used as negative controls. **(B)** The binding analysis of scFvs with subtype C 96ZM651 gp120 monomer. The anti-HIV-1 gp120 mAbs VRC01 and 447-52D were used as positive controls and mAb 2F5, HepB scFv, and mAb 1418 were used as negative controls. Mean binding titers were compared with negative control using unpaired *t*-test. *P* values <0.05 were considered as significant, (**) if *p* < 0.01 and (***) if *p* < 0.001.

**Figure 5:**
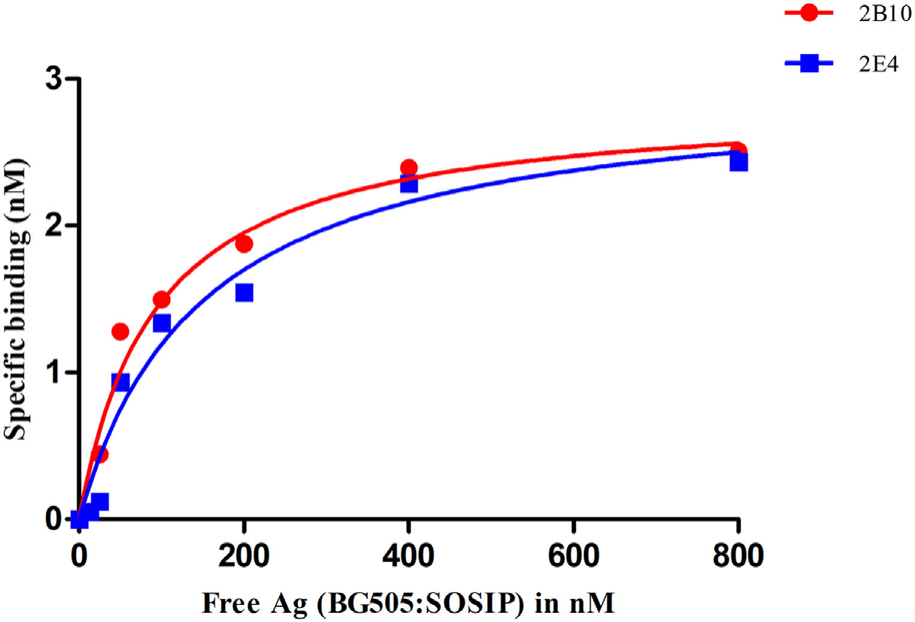
Analysis of the binding affinity of 2B10 and 2E4 scFvs by ELISA. Different dilutions of BG505:SOSIP.664 gp140 (10–800 nM) were incubated with fixed concentration of the two scFvs and unbound antibody was detected by ELISA. The experiment was repeated at least twice. Saturation binding curve was plotted between free antigen Vs specific binding through non-linear regression analysis.

The 2B10 and 2E4 scFvs were next tested for their ability to bind native-like HIV-1 trimeric envelope glycoprotein BG505:SOSIP.664 D-7324, and we observed high binding of both the scFvs with the trimeric antigen, which is known to bind only to bnAbs with high affinity and negligible binding with non-NAbs (Figure 4A). Further, binding analysis of both scFv monoclonals with recombinant HIV-1 96ZM651 subtype C protein confirmed their ability to bind with subtype C viral envelopes (Figure 4B). The affinity dissociation constant (KD) of both 2B10 scFv-BG505:SOSIP.664 gp140 complex and 2E4 scFv-BG505:SOSIP.664 gp140 complex was determined by ELISA and saturation binding curve was plotted through non-linear regression analysis (Figure 5). The Kd value for 2B10 scFv is 9.2 ± 1.4 × 10^−8^ M (R2 = 0.9709) and for 2E4 scFv is 1.4 ± 0.2 × 10^−7^ M (R2 = 0.9662). In binding analysis experiments, HepB, a scFv directed against hepatitis B antigen (54), was used as negative control and anti-CD4bs bnAb VRC01 was used as positive control.

### The 2B10 and 2E4 Anti-CD4bs Pediatric scFvs Exhibited Viral Cross-Neutralizing Activity

We assessed the neutralization activity of 2B10 and 2E4 scFvs against a standard panel of 49 pseudoviruses and primary isolates (7 subtype A, 13 subtype B, 24 subtype C, 1 subtype CD, 1 subtype AD, and 3 subtype AG) of different origin. The scFv monoclonals were tested at a starting concentration of 100 µg/ml with three-fold serial dilutions. The Geometric Mean Titers for the viruses neutralized by scFvs were calculated directly using MS-Excel. The 2B10 scFv monoclonal neutralized 38/49 viruses with GMT (Geometric mean titer) 17.9 µg/ml (20/24 subtype C, 9/13 subtype B, 5/7 subtype A, 1/1 subtype CD, 1/1 subtype AD, 2/3 subtype CRF02 AG) whereas, 2E4 neutralized 16/49 viruses with GMT 51.2 µg/ml (8/25 subtype C, 4/14 subtype B, 2/7 subtype A, 0/1 subtype CD, 0/1 subtype AD, and 1/6 subtype CRF02 AG) (Table 5). These results revealed better cross-neutralization breadth of the 2B10 scFv than 2E4; with 2B10 displaying cross neutralization across subtype C viruses from different geographical regions. The murine leukemia virus (MuLV) virus was used as negative control in all neutralization assays.

**Table 5:** Neutralization profile of anti-CD4bs scFv monoclonals 2B10 and 2E4.

VIRUS ID	SUBTYPE	TIER	ORIGIN	2B10	2E4	HepB
92RW020.2	A	2	Rawanda	<0.4	8.7	>100
B1206.W6P.ENV.A1	A	2	Kenya	41.4	>100	>100
BG505.W6M.ENV.C2	A	2	Kenya	30.9	68.1	>100
BJ613.W6M.ENV.E1	A	2	Kenya	8.44	>100	>100
BL274.W6M.ENV.A3	A	2	Kenya	28.3	>100	>100
Q168ENVa2	A	2	Kenya	>100	>100	>100
Q461ENVe2	A	2	Kenya	>100	>100	>100
6535.3	B	1B	USA	>100	>100	>100
AC10.0.29	B	2	USA	32.6	>100	>100
CAAN5342.A2	B	2	USA	26.8	>100	>100
JRCSF.JB	B	1	USA	10.0	41.57	>100
JRFL.JB	B	2	USA	3.64	52.6	>100
PVO.4	B	3	Italy	36.75	>100	>100
QZ4589	B	2	Trinidad and Tobago	>100	>100	>100
REJO4541.67	B	2	USA	>100	>100	>100
RHPA4259.7	B	2	USA	26.7	95.3	>100
SC422661.8	B	2	Trinidad and Tobago	>100	>100	>100
SF162.LS	B	1A	USA	9.92	24.8	>100
TRO.11	B	2	Italy	13.84	>100	>100
WITO4160.33	B	2	USA	63.8	>100	>100
001428-2.42	C	2	India	<0.4	39.6	>100
00836-2.5	C	1B	India	55.5	>100	>100
16055-2.3	C	2	India	38.25	88.58	>100
16936-2.21	C	2	India	36.4	>100	>100
25710-2.43	C	1B	India	<0.4	38.2	>100
25711-2.4	C	1B	India	26.8	82.5	>100
26191-2.48	C	2	India	32.75	>100	>100
CAP45.2.00.G3	C	2	South Africa	25.5	>100	>100
CAP210.2.00.E8	C	2	South Africa	>100	>100	>100
CAP256	C	2	South Africa	35.6	78.6	>100
DU156.12	C	2	South Africa	1.75	48.5	>100
DU172.17	C	2	South Africa	28.1	>100	>100
DU422.01	C	2	South Africa	<0.4	22.03	>100
MW965.26	C	1A	Malawi	7.58	>100	>100
ZM109F.PB4	C	1B	Zambia	80.2	>100	>100
ZM249.PL1	C	2	Zambia	69.5	88.63	>100
ZM53M.PB12	C	2	Zambia	70.5	>100	>100
AIIMS_329	C	2	India	26.4	92.2	>100
AIIMS_346	C	2	India	68.5	>100	>100
AIIMS_355	C	2	India	29.7	>100	>100
AIIMS_126	C	2	India	>100	>100	>100
AIIMS_201	C	2	India	>100	>100	>100
AIIMS_212	C	2	India	>100	>100	>100
AIIMS_254	C	2	India	64.8	>100	>100
BK184.W6M.ENV.D2	C/D	2	Kenya	28.3	>100	>100
BF535.W6M.ENV.A1	D/A	2	Kenya	36.8	>100	>100
33-7	CRF02_AG	3	Cameroon	72.13	96.41	>100
253-11	CRF02_AG	3	Cameroon	89.5	>100	>100
251-18	CRF02_AG	3	Cameroon	>100	>100	>100
Murine leukemia virus (MuLV)				>100	>100	>100

*The neutralization efficiency of 2B10 and 2E4 scFvs was tested with a standard panel of pseudoviruses and primary isolates of tier 1, 2, and 3. The scFv monoclonals were tested at starting concentrations of 100 µg/ml with serial threefold dilutions. HepB scFv was taken as negative control for scFvs. MuLV virus was used as a negative control. Non-linear regression curve straight line was plotted using the method of least squares to determine the IC50 values. Here, in this table, IC50 < 1 μg/ml is highlighted in red, 1 < IC50 < 10 μg/ml is in yellow, 10 < IC50 < 50 μg/ml is in leaf green, 50 < IC50 < 100 μg/ml is in faded green and IC50 < 100 μg/ml is in white*.

## Discussion

The recently isolated second generation anti-HIV-1 bnAbs using high throughput techniques such as antigen-specific single B cell sorting, memory B cell expansion, micro neutralization assays, and recombinant techniques exhibited near pan neutralization breadth (14) and have a promising potential to be used for passive immunization and gene therapy (17–22, 55, 56). One such bnAb that has cleared the safety and efficacy studies is VRC01, which targets the conserved CD4bs on gp120 and neutralizes HIV-1 by partially mimicking the binding of CD4 to this region (13, 22). Presently, VRC01 is being tested, in a number of human trails, for its safety, pharmacokinetics, and virological impact in infants at risk of infection and in HIV-1-infected aviremic and viremia subjects (20, 57). In addition to the therapeutic potential of whole IgGs, small antibody fragments (scFvs) are being evaluated as potential reagents for their protective effect (56, 58). The scFvs, by virtue of their small size, can gain access to sterically occluded epitopes that cannot be approached by whole IgGs (59–61). One of the targets of bnAbs, the CD4 induced (CD4i) site, is only transiently exposed on conformational changes following binding of the viral envelope gp120 to the CD4 receptor on immune cells. Recently and in the previous studies, the scFvs, targeting the CD4i site, exhibited broad neutralization potential with 80–100 percent breadth against the HIV-1 viruses tested from different subtypes globally (62–64).

The scFvs, such as 2B10, generated in this study, can be used as potential anti-HIV-1 reagents for inhibiting HIV-1 subtype C infection, by virtue of the ability to further engineer such recombinant antibody fragments to increase their potency and breadth, and affinity by site directed mutagenesis. However, there are chances that such modifications can also lead to reduction in the potency and affinity of scFvs. Furthermore, the short half-life of scFvs can be circumvented by fusing them with immunoglobulin binding domains as described previously (65). Modifications such as conjugation with the Fc portion of antibodies (59, 66) can also impart scFvs with effector functions such as antibody-dependent cell mediated cytotoxicity (ADCC), enabling them to better serve as reagents for passive immunization or gene therapy. Additionally, the scFvs with diverse epitope specificities can be used for the construction of bispecific antibodies that have the ability to bind two different epitopes on the viral envelope (67).

We recently reported the cross-neutralizing potential of two anti-CD4bs scFv monoclonals (D11 and 1F6) and one N332 V3-glycan directed scFv monoclonal (C11) identified from a human recombinant phage display library constructed from the PBMCs of HIV-1 sybtype C-infected adult cross-neutralizers. The CD4bs directed D11 scFv demonstrated 66% neutralization breadth and neutralized 33/50 viruses of different HIV-1 subtypes (43). In a recently conducted study, we observed the relative resistance of pediatric HIV-1 primary isolates to the existing bnAbs isolated from adults (33). HIV-1-infected children below the age of 2 if untreated, progress to AIDS, due to the immaturity of the immune system and limited exposure to diverse pathogens. Of late, however, cnAbs have been isolated from HIV-1 infected infants (31), providing us the impetus to generate a human recombinant scFv phage library and identify cross neutralizing CD4bs directed scFvs, using PBMCs from select pediatric cross-neutralizers. During the course of chronic infection, there is increase in breadth of the cnAbs induced by the diverse antigenicity of the circulating virus quasi-species (68–70). Our recent study on chronically infected children have also shown the presence of V1V2, V3, and CD4bs specific plasma antibodies in children, suggesting the development of bnAbs targeting multiple epitopes on HIV-1 envelope in these infected children (38).

The advantage of pooling the PBMCs from these cross-neutralizers is to obtain a recombinant scFv library of diverse epitope specificities with high probability of having scFvs with broad and potent neutralizing activity (43). Such libraries can be probed in future using newer HIV-1 antigens to identify scFvs directed at different regions on the envelope. It is less probable to get such potent bnAbs of diverse specificities from a single infected donor. Moreover, by way of generating recombinant antibodies, it is possible to get distinct antibody gene usages favoring breadth and potency that may not be seen to evolve in naturally infected donors. The plasma antibodies binding with RSC3 core protein at high titers and not with the RSC3 mutant Δ371I/P363N suggested the presence of VRC01-like antibodies in these pediatric donors that further increased the probability of identifying anti-CD4bs VRC01-like scFvs from the pediatric scFv phage library constructed in this study.

The scFv monoclonals 2B10 and 2E4 exhibited the same heavy chain gene usage of IGVH1-2, as is also shown by the anti-CD4bs VRC01 like bnAb lineage (13, 71). Also, unlike the anti-V1/V2 antibodies and V3-glycan antibodies, which have large CDRH3 lengths (8), the 2B10 and 2E4 scFv monoclonals have shorter CDRH3 lengths of 13 and 18 amino acid residues respectively, which is comparable with the CDRH3 lengths of VRC01-like antibodies (13). An interesting observation was the moderate frequency of VH nucleotide SHM, 10.1 and 11.1% in the scFv genes of 2B10 and 2E4, respectively, contributing to the neutralization breadth (77%) demonstrated by 2B10 scFv, suggesting the evolution of bnAbs in chronically infected children with higher SHM. Low levels of SHM (2–7%) have earlier been shown in infant derived antibodies with limited neutralization breadth (31).

Genetic variations are documented between viruses of different subtypes (inter-clade) and also within a subtype (intra-clade) (21, 23–26). A salient finding of this study is the ability of the 2B10 scFv to effectively neutralizing subtype C viruses (77%); from India and across subtype C viruses from other geographical regions, identifying this monoclonal as a potential therapeutic reagent. It is important to generate similar antibodies that have neutralizing potential across viruses of each subtype that can serve as a pool of protective antibodies. Further, the 2B10 scFv specifically neutralized all the subtype C and subtype A viruses tested in this study, isolated from pediatric subjects which were previously reported to be resistant viruses when tested against a panel of first and second generation adult bnAbs (33). Some of the factors that may be responsible for this could be the differences in the immune responses that evolve in children than adults and the immune selection pressure on the circulating viruses in them. It could also be because the likelihood of intra-clade differences is less than inter-clade differences. Perhaps in the future, antibodies isolated from pediatric donors, such as the 2B10 scFv that neutralizes subtype C pediatric viruses of different origin, may have an important clinical role in preventing infection in children. This is all the more important because most of the known bnAbs have originated from non-subtype C infected adults, whereas the disease burden is more with subtype C viruses.

To conclude, we have for the first time successfully generated a scFv phage library from HIV-1 subtype C chronically infected antiretroviral naïve children. From this library, we identified 2B10 as a cross-neutralizing CD4bs directed scFv monoclonal with reasonable breadth and potency, demonstrating neutralizing activity against tier 1, 2, and 3 viruses, including viruses of both adult and pediatric origin. Such anti-HIV-1 human scFvs, are potential candidates for conferring passive protection. Moreover, a combination of scFvs with antiretroviral drugs can be an effective strategy to suppress viremia at an early stage and thus block HIV-1 infection in children, mainly acquiring infection by vertical transmission.

## Ethics Statement

This study was approved by the Institute ethics committee, All India Institute of Medical Sciences (AIIMS), New Delhi, India (IEC/NP-536/04.11.2013), and all the experiments were carried out in accordance with relevant institutional and national guidelines and regulations. Written informed signed consent forms were obtained from the parents/guardians of all the study subjects.

## Author Contributions

KL designed and planned the study, edited and finalized the manuscript. SS provided scientific inputs in phage library construction and in discussion section of the manuscript. RL and SKK provided the HIV-1 infected pediatric donors sample. SK performed the research, data analyses, and wrote the manuscript. RK helped SK in construction of phage library; MAM generated the pseudoviruses for neutralization assays; RT, MM, and MA helped SK for the purification of scFv protein, LK helped SK in competition ELISA and affinity determination of scFv monoclonals.

## Conflict of Interest Statement

The authors declare that the research was conducted in the absence of any commercial or financial relationships that could be construed as a potential conflict of interest.
