# Supplementary material for: CD4-Binding Site Directed Cross-Neutralizing scFv Monoclonals from HIV-1 Subtype C Infected Indian Children
Source: Front Immunol. 2017 Nov 15;8:1568. doi: 10.3389/fimmu.2017.01568 (PMC5694743; doi:10.3389/fimmu.2017.01568)
Supplement: Supplementary file 1 [file data_sheet_1.pdf]

Supplementary Material

CD4-Binding Site Directed Cross-Neutralizing scFv Monoclonals from HIV-1 Subtype C Infected Indian Children

Sanjeev Kumar[1], Rajesh Kumar[1], Lubina Khan[1], Muzamil Ashraf Makhdoomi[1], Ramachandran Thiruvengadam[1], Madhav Mohata[1], Mudit Aggarwal[1], Rakesh Lodha[2], Sushil Kumar Kabra[2], Subrata Sinha[1] and Kalpana Luthra[1]*

*Correspondence: Prof. Kalpana Luthra: E-mail: kalpanaluthra@gmail.com

Supplementary Figures and Tables

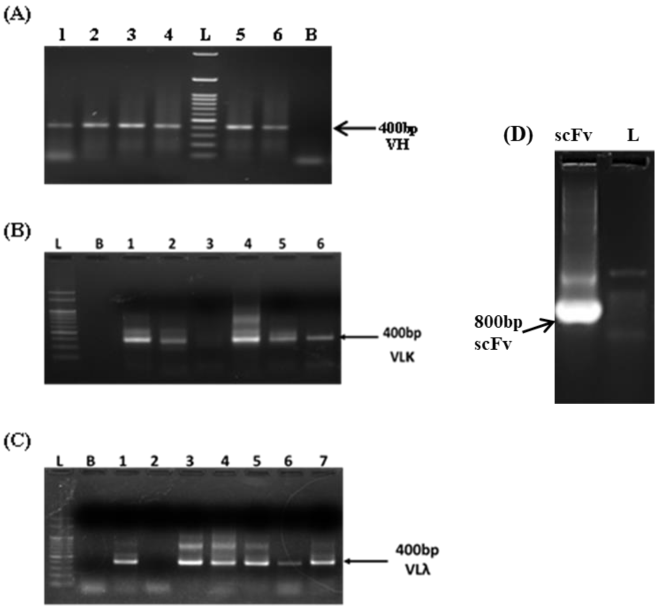

Figure S1. Amplification of variable heavy chain genes (VH) and variable light chain genes kappa (VLκ) and lambda (VLλ). Amplified PCR products were analysed on agarose gel (1.5%) electrophoresis. Lane B-NTC (non-template control). Lane L-100bp DNA ladder **(A)** Lanes 1-6 are amplified VH1, VH2, VH3, VH4, VH5 and VH6 genes respectively **(B)** Amplified VL kappa chain genes. Lanes 1-6 are amplified VLκ1, VLκ2, VLκ3, VLκ4, VLκ5 and VLκ6 genes respectively. VLκ3 was not amplified **(C)** Amplified VL lambda chain genes. Lanes 1-7 are amplified VLλ1, VLλ2, VLλ3, VLλ4, VLλ5, VLλ6 and VLλ7 genes respectively. VLλ2 gene was not amplified. **(D)** 800bp of scFv gene gene was amplified by pullthrough PCR.

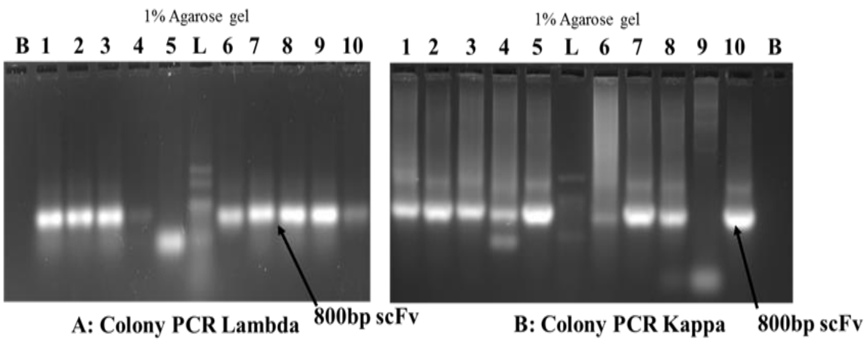

Figure S2. Colony PCR analysis of twenty randomly picked scFv clones before biopanning. 1% Agarose gel electrophoresis of colony PCR products of 10 randomly selected scFv clones containing **(A)** lambda light chain and **(B)** kappa light chain from unscreened phage library. Lane B-NTC (non-template control), Lane L-100bp DNA ladder. Lanes 1-10 are number IDs for randomly picked clones. One lambda scFv clone (lane 5) and one kappa scFv clone (lane 9) were not amplified.

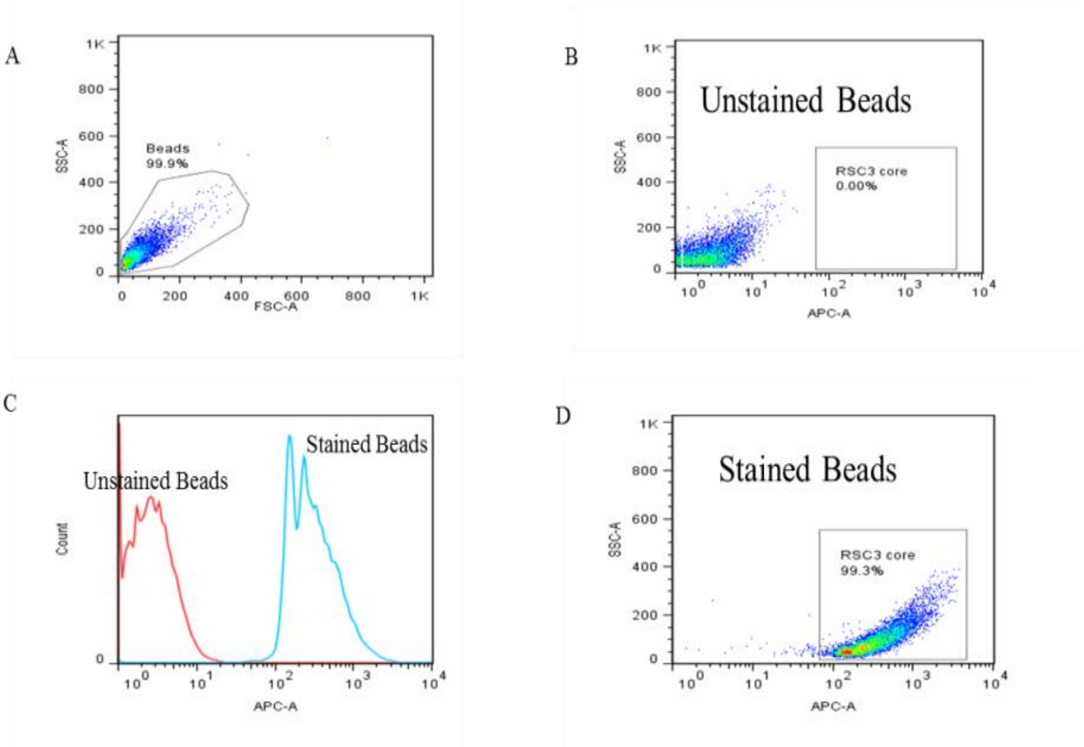

Figure S3. Flow cytometric analysis to determine coating of RSC3 core protein onto magnetic beads: (A) Gated 10,000 events. **(B)** Unstained beads used as negative controls. **(C)** Histogram representation of unstained and stained beads with RSC3 core identified by using bnAb VRC01. **(D)** Showing the percentage of APC stained RSC3 core protein coated magnetic beads.

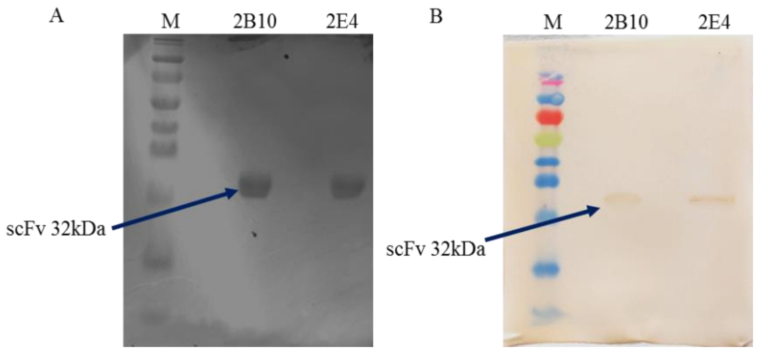

Figure S4. SDS-PAGE and Western blot analysis of purified scFvs 2B10 and 2E4. (A) SDS-PAGE (12%) analysis of purified scFvs. Gel was stained with Coomassie Brilliant Blue dye, Lane M, protein marker **(B)** Western blot analysis of purified scFv monoclonals. Lane M is prestained molecular weight marker. 32kDa scFv band was detected by mouse-anti-His-tagged antibody (1:1000) followed by secondary antibody anti-mouse-HRP (horseradish peroxidase) and DAB as substrate.

Table S1. Gene usage of randomly picked scFv clones to determine the diversity of the phage library. 18 colony PCR positive scFv clones were sequenced and their gene usage was analysed by using IMGT/V-Quest software. All 18 scFv clones were distinct. VH is variable heavy chain genes and VL is variable kappa/lambda light chain genes.

scFv ID	VH-Genes	VL-Genes
L 1	IGHV5-51*01	IGLV1-44*01
L 2	IGHV4-39*07	IGLV1-40*01
L 3	IGHV3-23*04	IGLV2-14*03
L 4	IGHV4-34*13	IGLV2-14*01
L 6	IGHV4-39*07	IGLV2-8*03
L 7	IGHV3-21*01	IGLV1-47*01
L 8	IGHV3-48*04	IGLV2-14*03
L 9	IGHV3-30*03	IGLV2-8*03
L 10	IGHV3-23*04	IGLV2-8*03
K 1	IGHV4-59*03	IGKV4-1*01
K 2	IGHV3-23*04	IGKV3-15*01
K 3	IGHV3-48*04	IGKV3-20*01
K 4	IGHV4-61*02	IGKV1D-43*01
K 5	IGHV3-21*01	IGKV3-20*01
K 6	IGHV3-33*01	IGKV3-20*01
K 7	IGHV4-39*07	IGKV3-15*01
K 8	IGHV3-48*04	IGKV2-24*01
K 10	IGHV4-59*01	IGKV1-5*03

Table S2. List of primers used for heavy chain and light chain antibody genes amplification for the construction of human scFv phage display library. Bold fonts indicate sequence complementary to the V-gene segments. Recognition sites for restriction enzymes (SfiI/NotI), and linker sequence are italicised. Modified from (1).

Primers	Sequence
V_H5'Sfi	5' CCTTTCTATGC*GGCCCAGCCGGCC* **ATGGCCCAGGTGCAGCTGGTGCAGTCTGG** 3'<br><br>5' CCTTTCTATGC*GGCCCAGCCGGCC* **ATGGCCGAGGTACAGCTGCAGCAGTCAGG** 3'<br><br>5'

	CCTTTCTATGC*GGCCCAGCCGGCC* **ATGGCCCAGGTCAACTTAAGGGAGTCTGG** 3'<br><br>5' *GCCCAGCCGGCC* **ATGGCCGAGGTGCAGCTGGTGGAGTCTGG** 3'<br><br>5' *GCCCAGCCGGCC* **ATGGCCCAGGTGCAGCTGCAGGAGTCGGG** 3'<br><br>5' *GCCCAGCCGGCC* **ATGGCCGAGGTGCAGCTGTTGCAGTCTGC** 3'
V_H3'link	5' *ACCAGAGCCGCCGCCGCCGCTACCACCACCACC* **TGAGGAGACGGTGACCAGGGTGCC** 3'<br><br>5' *ACCAGAGCCGCCGCCGCCGCTACCACCACCACC* **TGAGGAGACGGTGACCGTGGTCCC** 3'<br><br>5' *ACCAGAGCCGCCGCCGCCGCTACCACCACCACC* **TGAAGAGACGGTGACCATTGTCCC** 3'<br><br>5' *ACCAGAGCCGCCGCCGCCGCTACCACCACCACC* **TGAGGAGACGGTGACCAGGGTTCC** 3'
V_L5'link-κ	5' *AGCGGCGGCGGCGGCTCTGGTGGTGGTGGATCC* **GACATCCAGATGACCCAGTCTCC** 3'<br><br>5' *AGCGGCGGCGGCGGCTCTGGTGGTGGTGGATCC* **GAAATTGTGCTGACTCAGTCTCC** 3'<br><br>5' *AGCGGCGGCGGCGGCTCTGGTGGTGGTGGATCC* **GATGTTGTGATGACTCAGTCTCC** 3'<br><br>5' *AGCGGCGGCGGCGGCTCTGGTGGTGGTGGATCC* **GAAATTGTGTTGACGCAGTCTCC** 3'<br><br>5' *AGCGGCGGCGGCGGCTCTGGTGGTGGTGGATCC* **GACATCGTGATGACCCAGTCTCC** 3'<br><br>5' *AGCGGCGGCGGCGGCTCTGGTGGTGGTGGATCC* **GAAACGACACTCACGCAGTCTCC** 3'

V_L5'link-λ	5' *AGCGGCGGCGGCGGCTCTGGTGGTGGTGGATCC* **AATTTTATGCTGACTCA GCCCCA** 3'<br><br>5' *AGCGGCGGCGGCGGCTCTGGTGGTGGTGGATCC* **CAGTCTGTGTTGACGCA GCCGCC** 3'<br><br>5' *AGCGGCGGCGGCGGCTCTGGTGGTGGTGGATCC* **CAGTCTGCCCTGACTCA GCCTGC** 3'<br><br>5' *AGCGGCGGCGGCGGCTCTGGTGGTGGTGGATCC* **TCCTATGTGCTGACTCA GCCACC** 3'<br><br>5' *AGCGGCGGCGGCGGCTCTGGTGGTGGTGGATCC* **TCTTCTGAGCTGACTCA GGACCC** 3'<br><br>5' *AGCGGCGGCGGCGGCTCTGGTGGTGGTGGATCC* **CACGTTATACTGACTCA ACCGCC** 3'<br><br>5' *AGCGGCGGCGGCGGCTCTGGTGGTGGTGGATCC* **CAGGCTGTGCTCACTCA GCCGTC** 3'
V_L3'NotI-κ	5' CAGTCATTCTCGACTT*GCGGCCGC* **ACGTTTGATTTCCAGCTTGGTCCC** 3'<br><br>5' CAGTCATTCTCGACTT*GCGGCCGC* **ACGTTTAATCTCCAGTCGTGTCCC** 3'<br><br>5' CAGTCATTCTCGACTT*GCGGCCGC* **ACGTTTGATCTCCAGCTTGGTCCC** 3'<br><br>5' CTCGACTT*GCGGCCGC* **ACGTTTGATATCCACTTTGGTCCC** 3'<br><br>5' CTCGACTT*GCGGCCGC* **ACGTTTGATCTCCACCTTGGTCCC** 3'
V_L3'NotI-λ	5' CAGTCATTCTCGACTT*GCGGCCGC* **ACCTAAAACGGTGAGCTGGGTCCC** 3'<br><br>5' CTCGACTT*GCGGCCGC* **ACCTAGGACGGTGACCTTGGTCCC** 3'<br><br>5' CTCGACTT*GCGGCCGC* **ACCTAGGACGGTCAGCTTGGTCCC** 3'

PTfw	5' CCTTTCTATGC*GGCCCAGCCGGCC*ATGGCC 3'
PAK kappa *Sfi*	5' TCAGCAT*GGCCCCCGAGGCC*GCACGTTTRA T 3'
PAK lambda *Sfi*	5' TCAGCAT*GGCCCCCGAGGCC*GCACCTARRA C 3'

References

1. Pansri P, Jaruseranee N, Rangnoi K, Kristensen P, Yamabhai M. A compact phage display human scFv library for selection of antibodies to a wide variety of antigens. BMC biotechnology. 2009;9:6.
